# Practitioner perspectives on implementing a quality improvement intervention for telephone-based therapy in NHS Talking Therapies: a qualitative process evaluation study

**DOI:** 10.1186/s12913-026-14330-7

**Published:** 2026-03-20

**Authors:** Cintia Faija, Judith Gellatly, Kelly Rushton, Penny Bee, Helen Brooks

**Affiliations:** 1 Department of Primary Care & Mental Health, Institute of Population Health, University of Liverpool, Eleanor Rathbone Building, Liverpool, L69 7ZA UK; 2 Division of Nursing, Midwifery and Social Work, School of Health Sciences, Faculty of Biology, Medicine and Health, Manchester Academic Health Science Centre, University of Manchester, Jean McFarlane Building, Oxford Road, Manchester, M13 9PL UK

**Keywords:** Telephone therapy, Psychological therapy, Anxiety, Depression, Qualitative, CFIR, NPT, Implementation, Talking therapies

## Abstract

**Background:**

Understanding the factors that shape implementation processes is fundamental to improving outcomes and applicability of interventions. This study reports on part of a process evaluation nested within a cluster randomised controlled trial. It aimed to explore professional perspectives on the challenges and lessons learnt during implementation of a multilevel (patient, practitioner, and service-level) quality improvement intervention developed to enhance telephone-delivered psychological interventions in NHS Talking Therapies services for anxiety and depression (NHS TTad).

**Methods:**

Qualitative semi-structured interviews with twenty-eight professionals recruited from nine NHS TTad services were conducted. The Consolidated Framework for Implementation Research (CFIR) and Normalisation Process Theory (NPT) were used to inform data collection and analysis. Template analysis was used to exploit the structured approaches of CFIR and NPT whilst retaining the flexibility of thematic analysis to examine the factors perceived to affect implementation.

**Results:**

Four themes were interpreted from the data: (1) Perceived value of the intervention and its distinctiveness from routine practice; (2) Implementation climate and variation in uptake across services and practitioners’ telephone experience; (3) Need for implementation clarity and leadership support within existing constraints; and (4) Planning for monitoring and continuous improvement throughout implementation. Supportive leadership, planning and execution were important factors, supporting implementation into routine practice. Team members valued the protected time they had whilst in receipt of the intervention, reporting they could reflect on current practices, strengthen relationships and engage in collaborative feedback which supported co-creation of quality improvement plans to enhance telephone-delivered low-intensity psychological interventions. Analysis also revealed limited changes were implemented and sustained over time.

**Conclusions:**

Successful implementation and sustainability of the intervention across study sites was dependent on active leadership engagement, sufficient time and resources to build a shared understanding of work processes. Collective recognition of the value of the intervention by team members and clear understanding of what changes are needed, who is responsible for them, and the timeline for implementation was considered vital and may have implications for successful implementation nationally in Talking Therapy services. The COVID-19 pandemic significantly influenced the context of implementation, accelerating the adoption of telephone services but also reshaped service priorities and challenges during the study period.

**Trial registration:**

ISRCTN22583714, Registration date: 01/09/2021

**Supplementary information:**

The online version contains supplementary material available at 10.1186/s12913-026-14330-7.

## Background

In England, UK, over nine million people are diagnosed with a mental health disorder, with the vast majority experiencing anxiety and/or depression [1]. The prevalence of common mental health problems (anxiety and depression) is increasing, with over 20% of adults aged 16 to 64 in the UK experiencing one or both conditions between 2023-2024 [2] Consequently, mental health treatment access is increasing.

The impact of anxiety and depression at an individual and societal level is significant with negative impacts on social and occupational functioning and high healthcare costs [3]. Societal costs in the UK are estimated to be over £117.9 billion [4], with 9.8% of UK sickness absence attributed to mental health [5]. The UK aimed to address this burden by implementing a national Talking Therapies for anxiety and depression (NHS TTad) programme (formerly Improving Access to Psychological Therapies (IAPT) services) which aims to provide individuals with appropriate evidence-based psychological therapies in a timely manner using a stepped care approach [6]. Using this model, individuals are offered the least intensive, most appropriate treatment in the first instance (low intensity (step 2) interventions (i.e. guided self-help)) and are ‘stepped up’ to a more intensive treatment (high intensity (step 3)) if no benefit is gained. Interventions can be delivered using a variety of modalities including face-to-face, telephone and online treatment. More intensive treatment (Step 4) is offered to a smaller number of individuals for whom step 2 and/or step 3 treatments have not been sufficient, or who are experiencing severe and enduring complex mental health problems that require more intense specialist support.

Telephone as a modality offers a potentially more accessible and efficient approach to the delivery of psychological therapy [7, 8], 1.76 million referrals were made to NHS TTad services from 2022 to 2023 [9]. Historically, despite being acknowledged in national TTad training manuals, remote modalities have been inconsistently utilised across TTad services but mostly commonly used at step 2 [10]. Barriers to delivery include scepticism and resistance from patients and practitioners [7, 11] despite evidence indicating comparable efficacy between face-to-face and telephone [12]. These issues were further highlighted during the COVID-19 pandemic where all services moved to remote delivery necessitated by national lockdowns and social distancing policies [13–16]. Emerging evidence from this period indicates that there were no detrimental impacts on patient outcomes [13, 14] whilst also highlighting practitioner concerns about the lack of dedicated and evidence-based training which amplified their concerns about their capability to support patients optimally over the phone. [8–17].

## EQUITy intervention trial

The EQUITy (Enhancing the quality of psychological interventions delivered by telephone) research programme, funded by the UK National Institute for Health and Care Research (NIHR) aims to increase engagement in telephone-delivered psychological interventions for mild to-moderate depression and anxiety in TTad services (Step 2, low intensity), and improve clinical and cost-effectiveness. The programme has been guided by the four iterative phases of the Medical Research Council (MRC) framework for development and implementation of new and complex health care interventions [15] − 1. Intervention development, 2. Feasibility and piloting, 3. Evaluation, and 4. Implementation.

The first multi-component service-improvement intervention and implementation approach was designed (hereinafter referred to as the EQUITy intervention). Encompassing substantial pre-implementation work, it was derived directly from the views, experiences and observations from 78 stakeholder interviews (patient, practitioner, and local/national decision-makers) and analysis of 66 audio recordings of telephone-delivered assessment sessions conducted during the intervention development phase and refined during feasibility [7, 8, 11, 16]. Moreover, it drew upon theories of behaviour change [17] to optimise success.

The intervention comprised of three components of equal importance (further information about the intervention can be found in Appendix 1 and in an earlier paper describing its development and refinement [18]. It is also registered on the ISRCTN Registry (10.1186/ISRCTN22583714).

1. **Guidelines for services, team workshop and follow-up meetings** - Online day workshop for all members of the talking therapies step 2 team (including practitioners (trainee and qualified), service leads service managers and those involved in administrative roles. Attendees were introduced to the EQUITy programme and had the opportunity to identify low, medium and high priority areas for their service to enhance telephone-delivered therapies. Follow-up meetings with the research team 6-8 weeks post-workshop and subsequently bimonthly were offered to support implementation.
2. **Practitioner telephone training -** Following the workshop, two online 3-hour training sessions aimed to enhance practitioner telephone skills and reflect on their experiences of working over the telephone. The sessions focused on telephone skills development, including a mixture of interactive presentations, practical small group activities, audio/visual clips and live demonstrations of good practice.
3. **Resources for patients –** Three resources were co-designed and developed with the EQUITy Lived Experience Advisory Group (LEAP) - leaflet, appointment card and poster. They addressed issues and misconceptions about telephone treatment identified in early programme studies with patients and professionals, aiming to better help patients to understand and engage with, telephone therapy.

## Implementation approach

The EQUITy intervention was developed to enhance, rather than change the underlying principles of existing practice. The uniqueness of its approach was that it brought teams together to develop structured action plans based on the perceived needs of their individual service, introduced follow-up strategies to review implementation progress and success and incorporated service-user co-produced resources. The implementation approach emphasised the importance of leadership, individual and team empowerment and education to maximise effective organisation, execution and sustainability [19].

Implementation of the EQUITy intervention following attendance at the workshop included a variety of activities. Sites were informed they should implement their action plans as agreed. Knowledge and skills developed and attained during the sessions were to be implemented into the organisation and delivery of their usual telephone-delivered assessments and sessions with patients. Sites were also requested to distribute the patient resources to patients (electronically or by post) referred to telephone treatment. Action plan leads were informed they would be contacted by the Implementation Lead to arrange a 6–8-week post workshop follow-up meeting to discuss implementation success and identify strategies to overcome barriers faced if required.

The randomised controlled trial (RCT) in which the intervention was evaluated, and this this process evaluation was nested, was a cluster RCT where 11 of 21 of the recruited TTad sites received the intervention. There is increasing recognition that RCTs are complemented by a process evaluation. Understanding how interventions work, or do not work, in practice is fundamental to increasing their effectiveness and applicability, and developing evidence-based policy and practice [11]. Using the Medical Research Council (MRC) process evaluation model [20] to explore implementation processes, mechanisms and context, and informed by new standards for reporting implementation studies [21, 22], we conducted a qualitative interview study conducted with professionals from services allocated to the intervention arm of the trial, those tasked with implementation [23]. We aimed to explore experiences of intervention implementation, understand barriers/enablers to its roll-out and examine the extent to which practitioners deliver, and patients engage with, best-practice telephone-treatments. The study will augment the findings of the main trial (to be reported at a later date).

Interviews focused on exploring participant’s individual experiences of intervention implementation with regards to - the perceived impact of the EQUITy intervention (i.e. Service Guidelines and Team Workshops, Telephone Training and Patient resources) on professional development, patient experience and service performance; how the EQUITy intervention components have precipitated changes in service telephone delivery and; barriers and facilitators to the uptake and implementation of the intervention components and subsequent maintenance of changes in routine clinical practice.

This study is informed by a social constructionist perspective, which recognises that knowledge and meaning are co-constructed through language, social interaction, and context [24]. By adopting a social constructionist stance, this study seeks to make visible the ways in which practitioners make sense of and navigate implementation within their specific service contexts, acknowledging that these meanings are negotiated, contingent, and shaped by broader systemic influences.

## Methods

This study is one element of the wider process evaluation embedded in the EQUITy trial. It is a qualitative process evaluation involving interviews with professionals from services allocated to the intervention arm of the trial. Other elements of the process evaluation included interviews with intervention arm patients and pre-post intervention data collected from practitioners attending the team workshop and/or practitioner training elements of the intervention. A podcast, co-produced with the EQUITy Lived Experience Advisory Panel, was created to summarise patient interview findings (https://www.youtube.com/watch?v=csx_-PWZejc). Data pertaining to pre-post data will be presented in the main trial paper. Reporting is informed by the Consolidated Guidelines for the Reporting of Qualitative Data (COREQ) [25]. Ethical approval was granted by the North West—Preston Research Ethics Committee (REF: 21/NW/0218; IRAS ID: 298,615). Research governance was approved at all participating NHS sites and participants provided informed consent online prior to participation.

### Sampling

Recruitment took place from March 2022 to June 2023. TTad service professionals in services who attended the EQUITy intervention workshop and/or telephone training sessions were invited to take part in an interview or a group discussion 16–24 weeks after attending the initial workshop. They were sent an information pack by the EQUITy team via email, which included an invitation letter, an information sheet, and a consent to contact form. The information sheet provided details about who would conduct the interviews/group discussion and their aim/purpose with respect to the immediate study and the wider EQUITy programme. Participants were given as much time as they needed to decide whether to participate (within the constraints of the trial). Those who were interested, could email the research team to express their interest or complete and return a consent-to-contact form by email. If they remained interested after this initial contact, a convenient date/time to participate was arranged.

We purposively sampled across all the professionals in the intervention sites who attended the training and workshops as part of the EQUITy intervention to ensure variance in clinical and organisational contexts. There were no exclusion criteria. A sampling frame captured information about professionals’ organisations, roles (e.g., practitioners, service managers, service leads), gender, the number of intervention sessions attended, and the level of experience in their current role.

### Data collection and management

The interview schedule, developed specifically for this study, was informed by the Normalisation Process Theory (NPT) [26] and the Consolidated Framework for Implementation Research (CFIR) [23] (see supplementary file 1). These were adopted to ensure maximum value of and relevance of interview data. NPT focuses on the work people need to do to implement, embed and sustain interventions in existing healthcare practice, while the CFIR is a determinant framework commonly used in the development and evaluation of complex interventions. It comprises a taxonomy of 39 operationally defined constructs across five domains which influence the implementation of complex interventions. The domains relate to the planned intervention, the contexts in which the implementation activities will occur (known as the ‘inner’ and ‘outer’ settings), the individuals involved, and the process of intervention delivery. We used the theories as an organising lens rather than a rigid structure to guide data collection and analysis following appraisal of the different approaches available [27]. This approach allowed for emergent themes to be identified whist still providing structure. The development of overarching interpretative themes was the final stage of our analysis process.

Using these in conjunction can help illuminate a more comprehensive understanding of the interplay between the work that people must do to successfully implement an intervention and the context in which they are based during implementation [28].

Although potential participants were offered the opportunity to take part in an individual interview or group discussion all chose the option of an interview. Individual interviews (*n* = 28) were conducted by telephone, by an experienced female qualitative researcher who has practiced as a psychologist (CF). Participants had attended a workshop/training session that the interviewer may have helped to facilitate but did not know them personally, nor were they aware of the interviewer’s perceptions of telephone therapy. Interviews lasted between 24 and 65 minutes (average length 45 minutes) and were audio-recorded with the participant’s consent on an encrypted device, then transcribed verbatim by a university-approved company. Any identifiable information was removed from the transcripts to protect participants’ anonymity.

### Data analysis

Data analysis was conducted blind to trial outcomes to avoid biased interpretation [22]. Transcripts were checked for accuracy by one researcher and then anonymised prior to allocation. Analysis followed a template analysis approach informed by NPT and CFIR frameworks to inform a thematic analysis of the interview data [29].

Transcripts were first read and then re-read to ensure sufficient familiarity with the data. Template analysis was used in the initial stages of data analysis to identify and organise themes [30] prior to mapping onto the CFIR and NPT frameworks respectively to develop a final template, maintaining closeness to the participants experiences and contexts prior to applying the frameworks to provide granularity. The template was refined by removing redundant framework components. Finally, the template was used to support the development of overarching interpretative themes which were considered to reflect the most salient and important aspects of professionals’ experiences during the implementation process. Analysis occurred in parallel to data collection to allow for comparison of experiences between different organisational contexts. Data analysis was facilitated using NVivo software [31].

An experienced qualitative researcher (CF) analysed the data. A proportion (10%) of the transcripts were coded independently by another experienced qualitative researcher (KR) to ensure reliability and accuracy of data interpretation. No major discrepancies were identified. Regular meetings took place with the process evaluation lead (HB) and the programme manager (JG) to discuss and reflect on the findings, potential biases, assumptions, and positions in relation to the research topic. Early discussions held involved operationalising NPT/CFIR components for our specific context (e.g. defining inner/outer setting) to promote shared understanding and ensure they were meaningful, interpretable and applicable to our specific settings and processes under examination [27]. To ensure trustworthiness of the qualitative findings, an audit trail was maintained throughout the analysis process, documenting decisions related to code development, framework mapping and agreement of concept meeting within the context of our study, theme refinement, and the reduction of redundant framework components. This allowed for transparent tracking of analytic steps. Emerging themes were reviewed and discussed within the research team to enhance credibility and reduce individual bias.

CF acknowledges that interpretations are shaped by her background as a practitioner without experience of working in NHS TTad services and as a researcher committed to improving patient care and service delivery via quality improvement initiatives. All researchers recognised the complex social, organisation and relational processes that influence change. Researchers view participants account not as objective representations of a single reality, but as situated narratives shaped by their roles, values, histories, and the contexts in which they work.

### Patient and public involvement

A lived experience advisory panel (EQUITy LEAP) collaborated with the research team at all stages of the project. KR was the researcher leading and facilitating meetings with the LEAP who contributed to the design, reviewed all participant-facing material, provided feedback on the interview schedule and piloted the interview prior to the first participant interview.

## Results

### Sample

One hundred and one professionals who attended the workshop and/or training sessions in the intervention arm were invited to participate, 33 expressed an interest, with 28 professionals across nine TTad services consenting to take part (Table 1). Despite numerous attempts to recruit, no professionals from the final two sites opted to participate. The aim was to recruit 2–3 professionals per intervention site (min 22; max 33); the study therefore surpassed the minimum target and reached 85% of the maximum target. Professionals included TTad service Psychological Wellbeing Practitioners (PWPs; senior, qualified and trainees), a High Intensity (HI) Therapist, Service Leads, Team Managers and non-clinical personnel.

**Table 1:** Participant characteristics

Site	Gender	Role	Time in role
9 sites	Female (19) Male (8) Prefer not to say (1)	PWP (11) Trainee PWP (2) Senior PWP (7) HI Therapist (1) Clinical Lead (4) Non-clinical (3)	Less than 1 year (14) 1 to 5 years (7) More than 5 years (5) No answer (2)

A concurrent analysis of the data collected indicated that sufficient information power [32] had been achieved. The interviews were of high quality, generating rich and focused data. The interviewer was responsive to participants’ narrative, reflecting effectively upon the content of the conversation, adapting their questioning in response to evolving accounts. Thus, pursual of participants in the final two sites did not continue.

Demographic characteristics of participants is presented in Table 1.

**Fig. 1:**
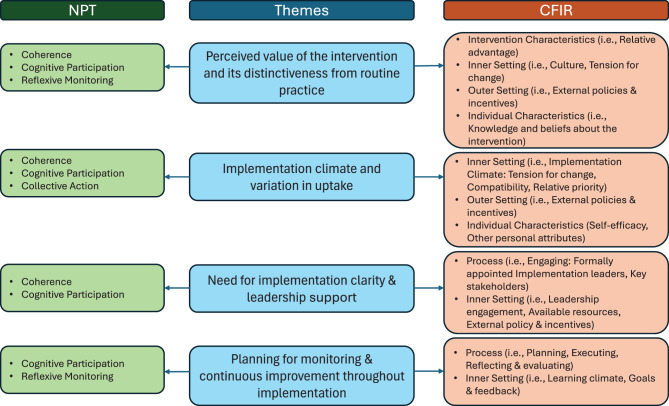
Salient NPT and CFIR themes and how they map to identified themes

The findings from both frameworks were integrated and are presented across four thematic areas, each combining aspects of CFIR and NPT, as detailed in Fig. 1:

(1) perceived value of the intervention and its distinctiveness from routine practice; (2) Implementation climate and variation in uptake across services and practitioners’ telephone experience; (3) Need for implementation clarity and leadership support within existing constraints; and (4) Planning for monitoring and continuous improvement throughout implementation.

#### Perceived value of the intervention and its distinctiveness from routine practice

This theme captured participants’ perceptions of the added value of the EQUITy intervention. Practitioners and service leads identified several benefits, including: (1) improved patient outcomes such as increased recovery rates, attendance, and engagement; and (2) enhanced opportunities for professional development, and a noticeable increase in staff morale. These gains were supported by structured time for reflection, opportunities for shared learning, and greater clarity around the use of telephone-based interventions, which together fostered a sense of professional growth, confidence, and team cohesion.

*I would hope that that’s the case anyway, you know, because if we’re delivering more effective treatment then we’re more likely to get better outcomes, which were better engagement within the service. (PWP05)*

*And just generally when you give CPD or any kind of training to staff members, it should improve the morale of the staff which is good for the service as well. (PWP01)*

Additional perceived benefits were participants’ valued engagement in research as a means of improving service performance, enhancing patient satisfaction, and contributing to the evidence base for telephone-delivered talking therapies at Step 2 care. This was considered particularly important given the perception that telephone therapy is sometimes viewed as a “*lower*” or “*second-class*” form of care compared to face-to-face therapy. The intervention appeared to increase perceived credibility of telephone treatment, a shift from early EQUITy studies that found telephone was perceived to be inferior to face-to-face sessions [7, 11]. This was further reported to be enhanced by a shift in societal and professional views about telephone therapy resulting from the COVID-19 pandemic. This shift contributed to improved acceptance and perceived legitimacy of telephone-based interventions, creating a more supportive environment for the implementation of telephone-based therapies in routine care. As one practitioner reflected:

*COVID-19 changed the way we view telephone therapy. It’s now more accepted, and that has made a difference in how we deliver care*. (PWP03)

Some practitioners and service leads highlighted the advantage of the patient follow-up data collected by the research team as part of the wider trial, noting that they believed it could help services monitor and evaluate performance over time—an activity not routinely undertaken.

The intervention was reported as being closely aligned with existing telephone-based practices and reinforced practitioners’ existing knowledge and skills. However, because the intervention built upon rather than transformed current practice, some participants questioned its novelty and potential impact. Further data illuminated that greater clarity to help distinguish the EQUITy intervention from routine practice would to be important to strengthen understanding about what is required and getting key individuals involved.

In summary, while the EQUITy intervention was valued for its perceived benefits to patient outcomes, practitioner development and service improvement, findings highlighted the need for greater clarity around its distinctiveness from routine practice to maximise engagement and uptake for consistent implementation.

#### Implementation climate and variation in uptake across services and practitioners’ telephone experience

Findings demonstrated that services involved in the trial were increasingly committed to delivering therapy by telephone, particularly following the COVID-19 pandemic. This commitment created external pressure to demonstrate effectiveness of interventions delivered by telephone, with practitioners and service leads recognising the importance of research and evaluation to secure ongoing funding:

*We pride[ourselves] a lot in trying to get research and trying to just understand what’s working and maybe what’s not working. (PWP03)*

The EQUITy intervention benefited from strong compatibility with existing telephone practices. It aligned well with practitioners’ established ways of working, facilitating its integration into routine workflows, reducing resistance to change, and enhancing engagement. Services with extensive experience in telephone-based interventions found EQUITy relatively easy to adopt, often requiring only minor adjustments. However, in these settings, the intervention was sometimes perceived as reinforcing existing practices rather than offering transformational change:

*I think that it is quite straightforward in terms of everything is in line, I think the way that it’s been created [the EQUITy intervention] is very much to support the way we work already as PWPs. (PWP07)*

Many practitioners viewed the intervention as enhancing their confidence in delivering therapy by telephone, rather than radically altering their practice, emphasising subtle shifts in confidence rather than overt behavioural changes:

*I’ve noticed a real vast improvement in my confidence with delivering the interventions now. (PWP03)*

However, engagement waned over time. Following the initial training period, motivation to deliver EQUITy-related practices declined for some practitioners. Some reported a sense of disillusionment, feeling that the intervention largely repeated what they were already doing:

*The clinicians were deflated after the training. It felt like we were already doing most of what the intervention suggested. (TM08)*

Practitioner characteristics also influenced the uptake and integration of the intervention. Less experienced practitioners were generally more enthusiastic and more likely to adopt changes, whereas more experienced staff tended to be less engaged and demonstrated minimal behavioural changes in their established practices.

Overall, the EQUITy intervention’s alignment with existing telephone delivery practices supported its integration into routine care. However, service- and practitioner-level differences in experience led to varied uptake, with experienced practitioners often perceiving limited added value. These findings highlight the importance of carefully assessing existing practices and flexibility in implementation strategies, ensuring that interventions are tailored to meet the needs of services and practitioners with varying levels of telephone delivery experience.

In summary, while the EQUITy intervention was generally well-aligned with routine telephone practices and supported practitioner confidence, its impact varied depending on existing service experience and practitioner engagement, highlighting the importance of tailoring implementation to local context and individual needs.

#### Need for implementation clarity and leadership support within existing constraints

The findings highlighted a critical lack of clarity around expected clinical changes in routine practice following the EQUITy intervention and processes specific to the trial. Practitioners were uncertain about how to implement the intervention components, including changes after training and the use of the EQUITy patient resources. Many expressed confusions and the absence of clear, formal plans for integrating the intervention into practice, which undermined readiness for implementation.

*I believe there was a lack of clarity on what was expected from us as practitioners. (SPWP09)*

This lack of clarity resulted in inconsistent distribution and use of patient resources across services. Practitioners often reported not knowing who was responsible for the various intervention elements, highlighting weaknesses in planning, monitoring, and role assignment.

In contrast, active leadership engagement emerged as a major facilitator of successful implementation of the action plan. Services where senior leaders championed and supported the EQUITy Action Plans demonstrated stronger commitment and more sustained efforts to embed the changes into routine practice. Examples of leadership involvement included attending the EQUITy workshop, co-developing action plan objectives with staff, allocating protected time for implementation activities, and monitoring progress in regular team meetings as well as with the research process evaluation lead. Such visible and ongoing support signalled the importance of the intervention and encouraged team-wide engagement.

*Having senior staff involved in the action plan made a big difference. Without that, it felt like a box-ticking exercise. (PWP05)*

The appointment of implementation champions, responsible for advocating the implementation of agreed action plans and maintaining contact with the research process evaluation lead—whether senior or junior staff—was key in maintaining momentum of the implementation process. Leadership presence during EQUITy workshops created a culture that legitimised and reinforced the intervention.

However, workforce constraints and external pressures including limited protected time for training and implementation activities, financial constraints, staff sickness, and high staff turnover often hindered implementation efforts by reducing staff availability to attend training, limiting opportunities for implementation planning, and disrupting continuity in the implementation of the EQUITy intervention. Furthermore, in some services, staffing gaps meant that implementation responsibilities were deprioritised in favour of meeting clinical demands. This affected uptake and created delays in embedding planned changes into routine practise, ultimately resulting in a loss of momentum.

Larger services faced challenges in ensuring all staff participated in the EQUITy training, due to the scale and complexity of their teams. In contrast, smaller services found it easier to engage practitioners and ensure more consistent attendance at training.

*Only about half of our Step 2 practitioners took part in the training, and since then we’ve recruited new staff who didn’t take part. (PWP01)*

In addition, no formal strategies were implemented to ensure that staff who were unable to attend the EQUITy training, including newly recruited staff, received the same training. This negatively impacted the sustainability of the intervention over time.

Moreover, some practitioners perceived the intervention as an added burden rather than a seamlessly integrated component of their routine practice.

*It just felt like another thing on top of everything else we already do.* (PWP12)

Overall, successful implementation of the EQUITy intervention was facilitated by leadership engagement and its integration with existing practices, but sustained uptake was challenged by unclear expectations, high staff turnover, limited resources, and competing service priorities. This highlights the likely importance of providing structured support, ongoing leadership involvement, and clearer communication of practitioner roles and responsibilities.

#### Planning for monitoring and continuous improvement throughout implementation

Findings highlighted that while practitioners recognised the potential benefits of the EQUITy intervention—such as increased patient satisfaction, treatment adherence, and recovery rates—the absence of a structured internal evaluation and follow-up strategy limited the ability to track and sustain its long-term impact. Over time, this lack of ongoing monitoring and feedback contributed to a gradual disengagement from the intervention.

The importance of establishing feedback loops and structured review mechanisms during implementation was emphasised. Data analysis revealed that although services monitored standard Key Performance Indicators (KPIs) (e.g., recovery rates), they did not consistently evaluate the specific effects of the EQUITy intervention.

*I monitor the KPI, so we do look at the different rates, like telephone compared to face to face […] I probably need to look at that a bit more in depth. (APL12)*

During interviews, practitioners spontaneously proposed clinical service activities to assess the intervention’s impact, such as conducting periodic reviews of patient dropout rates, recovery outcomes, and patient feedback:

*I guess maybe reviewing things like periodically throughout the year so having reviews, making clinical skills with other PWPs about having it as like a check-in point a few times in the year of how telephone work is going, any changes but also looking at things like dropout rates over the phone especially.* (PWP13)

Another practitioner similarly noted the potential for comparative evaluation:

*And you can compare maybe this feedback after receiving the EQUITy intervention with maybe feedback you have from previous years just to see if the patient experience has changed somehow.* (PWP02)

The lack of proactive planning to monitor and provide feedback to newly recruited services on the impact of the EQUITy intervention reflected missed opportunities to adapt and refine its implementation. Regular debriefs, clinical discussions, and structured follow-up meetings were identified as potential mechanisms to support shared learning, improve fidelity to the intervention, and tailor the approach to emerging needs.

Data also highlighted areas where clearer guidance would have supported better engagement and implementation. Issues with intervention packaging—such as the complexity of the first workshop, the user-friendliness of the “Service Guidelines Booklet,” and lack of clarity around expected practice changes—were identified as barriers to full integration into practice.

*I believe the general presentation and idea about the training was well delivered, but it left some confusion about what comes next. (SPWP09)*

Moreover, the lack of informal and formal supports for sustaining engagement—such as reminders, shadowing, and ongoing reinforcement—meant that over time, the EQUITy intervention was overshadowed by caseload pressures and competing service priorities:

*I think sometimes with caseloads going on and everything it can be difficult to stay on top of new trainings or new resources. (PWP07)*

Other data suggested that without ongoing organisational commitment to reinforce the value of the EQUITy intervention, initial enthusiasm diminished, and the intervention risked becoming deprioritised within routine practice.

Overall, these findings provide insight into the importance structured monitoring, regular feedback, and opportunities for continuous reflection and adaptation potentially have in sustaining practitioner engagement and ensuring the long-term success of complex multi-component interventions like EQUITy. Building formal and informal reflection mechanisms into service workflows could allow services to better measure impact, adapt implementation strategies over time, and maintain the intervention’s relevance alongside routine demands.

## Discussion

This study explored the implementation of the EQUITy intervention, aimed to enhance the quality of telephone delivery at Step 2 in NHS TTad services. The CFIR and NPT were used to develop a comprehensive understanding of the key factors influencing its integration into routine practice. Overall, findings indicate that while the EQUITy intervention was broadly compatible with existing telephone-based practices and initially well-received by services, significant challenges persisted in embedding its components into everyday care. The data revealed misalignment between perceived intervention value, organisational readiness, leadership engagement, and practitioners’ motivation, contributing to variation in uptake and sustained engagement. Despite initial enthusiasm, limited structured monitoring, unclear expectations, inconsistent leadership support, and workforce issues, including high staff turnover and heavy workloads, ultimately hindered the intervention’s long-term embedding and adaptation. High staff turnover posed a critical threat to sustainability, as the pool of trained personnel diminished over time. These findings reflect broader difficulties commonly encountered in translating research-led interventions into everyday practice, particularly within resource-constrained mental health settings [33]. Our findings identified key priorities critical to mental health and other contexts identified in pervious literature [34–36]. More specifically, they are affiliated with the key priorities that talking therapies workforce strategies highlight to enhance patient and staff experiences such as compassionate leadership and collaborative team working [37]. They additionally closely align with constructs from the CFIR and NPT. For example, the need for implementation clarity, consistency, and leadership support reflects CFIR’s Inner Setting domain (leadership engagement, implementation climate) and NPT’s emphasis on collective action and reflexive monitoring. Rather than introducing new constructs, our data provide empirical support for these frameworks and reinforce their applicability in mental health care settings, offering practical examples of how established constructs operate in contemporary real-world implementation.

The importance of leadership and communication strategies identified that promote clarity and consistency, aligns with prior implementation research. Leadership behaviours such as clear, consistent messaging within and across organizational levels and structured communication processes (e.g., regular team meetings, feedback loops, written updates) are critical for creating a positive implementation climate and supporting the uptake of evidence-based practices [33].

The context of the COVID-19 pandemic represents a critical backdrop to understanding the implementation and perceived novelty of the EQUITy intervention. The pandemic precipitated an unprecedented shift towards remote and telephone-based mental health service delivery, dramatically accelerating the adoption of digital and telephonic modalities across NHS Talking Therapies services [38, 39]. This rapid change has fundamentally altered the landscape of care, meaning that the pre-pandemic needs assessments, extensive stakeholder reviews, and service redesigns that informed the development of the EQUITy intervention may now require re-examination or adaptation to remain relevant. Many practitioners indicated that some elements of the intervention overlapped with evolving practice adaptations already implemented during the pandemic, challenging the perceived novelty and fit of the intervention within existing workflows. Recent studies highlight how mental health services have struggled to sustain quality and engagement in remote care, with concerns around staff capacity, digital inequalities, and the need for ongoing professional support to deliver effective telephone-based interventions [40–42]. Thus, while the EQUITy intervention aligns with the ongoing shift towards remote care, its successful implementation must consider the accelerated systemic changes and resource constraints induced by the pandemic. This underscores the importance of flexible, contextually sensitive implementation strategies that can evolve alongside the rapidly changing service delivery environment.

Implementation barriers and facilitators suggest six recommendations for future intervention design, delivery, and evaluation can be drawn attention to:

### Aligning with organisational capacity

Implementation efforts must be realistically aligned with available organisational capacity. Resource constraints—including limited time, high staff turnover, and reduced access to training materials—emerged as significant barriers to implementing the EQUITy intervention. These challenges were particularly evident in services organisations experiencing ongoing workforce instability and intense pressure to meet service targets. To prevent overwhelming services, a staged approach to implementation, as reflected in the action plan’s “plans for now, short term and long term,” can help spread the workload and allow for the gradual embedding of changes. Additionally, ensuring training accessibility by recording sessions and making them available to staff unable to attend or to new team members is critical to support ongoing learning and sustainability. Future implementation strategies should include thorough assessment of service readiness and tailor rollout plans accordingly, ensuring interventions are introduced when sufficient staff capacity, time, and support structures are in place.

### Recognising practitioner-specific contextual factors

Variation in practitioners’ prior experience with telephone interventions significantly influenced the degree of intervention uptake across services. More experienced practitioners, who were familiar with telephone delivery, perceived elements of the EQUITy intervention as redundant due to overlap with their established practices. In contrast, less experienced practitioners found elements of the EQUITy training revealing, which positively impacted behavioural change. These findings underline the importance of tailoring implementation strategies to individual practitioner contexts, considering differences in skills, confidence, and familiarity with telephone-based delivery to optimize engagement and effectiveness.

### Building supportive networks

Opportunities for informal debriefing, peer support, and collaborative reflection were highly valued by practitioners and appeared crucial for sustaining engagement. The informal debriefing that occurred spontaneously in some services following receipt of the intervention was useful. Participation in team reflection and peer support activities in the training and workshop was valued by practitioners. It fostered further collaborative teamwork after receipt of the intervention, something that was previously reported as lacking prior to their involvement, mainly due to restrictive working environments such as large, shared offices or individual home working. These findings align with broader evidence that peer learning, social networks, and distributed leadership enhance intervention sustainability [43]. Services should formally embed peer learning opportunities—such as group supervision, shadowing sessions, or peer mentorship—into their implementation plans. Building an organisational culture that promotes regular social learning will support motivation, adaptation, and collective ownership of change.

### Promoting clarity and consistent communication

A clear and compelling rationale for the intervention must be communicated to all stakeholders from the outset. Findings suggested that unclear expectations and perceived lack of novelty led to a decline in practitioner motivation over time. It is essential to explicitly communicate what specific practices need to change, how success will be measured, and why these changes matter for patients and services. Ensuring role clarity, outcome ownership, and ongoing dialogue about progress will be vital for promoting implementation fidelity and practitioner engagement [44].

### Structured planning, monitoring, and feedback loops

The absence of systematic planning and monitoring processes was a major barrier to long-term intervention sustainability. Services should embed clear implementation plans, with defined goals, timelines, responsibilities, and review points. Regular feedback loops—such as quarterly data reviews or team check-ins—could help practitioners adapt strategies based on real-time learning, maintain momentum, and build a culture of continuous improvement. Reflexive monitoring, as described by NPT, must become a core feature of ongoing practice to ensure that adaptations are responsive and grounded in evidence.

### Strengthening leadership and accountability

Leadership support emerged as a critical enabler of implementation success. Services with actively engaged senior leaders who were explicitly tasked with overseeing implementation—demonstrated higher levels of practitioner motivation and follow-through. Assigning named “implementation leads” and building clear lines of accountability at both service and team levels could help maintain momentum. Leadership commitment must extend beyond mere endorsement; it requires visible, active participation to align resources, protect time for training and reflection, and ensure the ongoing implementation of the EQUITy intervention as a service priority.

## Strengths and limitations

This study offers valuable insights into the practical challenges and enablers of implementing telephone-based interventions, utilizing well-established frameworks (CFIR and NPT) to identify key factors that appear to influence success. The use of in-depth qualitative interviews with a range of practitioners and service leads ensures a comprehensive understanding of the intervention’s implementation processes and highlights important considerations for future implementation efforts.

Using NPT alongside the CFIR provided a comprehensive understanding of the implementation process of the EQUITy intervention. While CFIR illuminated the contextual factors influencing uptake, NPT focused on the social and cognitive mechanisms that supported or hindered the intervention’s normalisation into practice.

This study warrants discussion of four main limitations. First, the variability in the execution of the different elements of the intervention across services, as well as inconsistent engagement from leadership and practitioners, raises important questions about consistency in implementation. The variability in implementation could affect how successful or effective the intervention appears in practice. Secondly, participants’ responses about their experiences, attitudes and behaviours towards the EQUITy intervention might be influenced by social desirability or memory recall issues as the interviews were purposively conducted at least 16 weeks following the receipt of the intervention to explore implementation facilitators and challenges. Thirdly, in analysing the interviews only a small proportion were double-coded. While this may be considered as a limitation, we acknowledged the introduction of potential bias during monthly analytic meetings with wider research team members that provided ongoing opportunities for reflexive discussion and validation of interpretations. Finally, this study focused on the views of practitioners and service leads regarding the implementation and perceived impact of the intervention. Given the subtle changes reported by professionals’ post-intervention and the fact that the intervention was not directly targeted at patients-, this perspective alone may offer limited insight into its broader impact. As such, potential effects on patient care and satisfaction were not explored in this paper.

## Future research

Building on these findings, future research should investigate the long-term sustainability of telephone-delivered interventions, tracking how implementation adapts over time in response to changes in the workforce and policy. Comparative studies examining different training delivery methods (e.g., workshops, e-learning, simulation) could identify the most effective approaches for building and maintaining telephone therapy skills. It would also be valuable to investigate tailored training needs for newly qualified versus experienced practitioners. Finally, future work should evaluate whether behavioural prompts, reminders, or incentives can enhance engagement and sustain practice change over time.

The contextual barriers identified in our analysis such as lack of supportive leadership, structured planning, and clear communication could be addressed by drawing on published recommendations such as the Expert Recommendations for Implementing Change (ERIC) project strategies, a taxonomy of 73 approaches for implementing change [45]. For example, strategies like developing a formal implementation blueprint, identifying and preparing champions, and building coalitions provide structured approaches to strengthen leadership engagement and planning, reducing ambiguity such as that observed in our study. Similarly, strategies such as developing and distributing educational materials, conducting ongoing training, and providing consultation directly target communication gaps could help ensure implementation clarity within and across organizational levels. Future research should examine the effectiveness of these approaches in mental health care settings.

## Conclusion

Findings highlighted key factors influencing the implementation of the EQUITy intervention. Key challenges included leadership engagement, workforce capacity, and staff motivation, while the adaptability of telephone interventions and practitioner training were crucial for successful implementation. Both frameworks emphasized the inclusion of clear action plans, consistent monitoring, sustained leadership support, and ensuring that training and support structures are adaptable to different levels of practitioner experience may enhance intervention implementation success. The COVID-19 pandemic significantly altered the landscape of telephone-based services, accelerating their adoption but also shifting pre-existing needs and priorities, which impacted the relevance and fit of the intervention during the study period. Policymakers and service leads should consider embedding structured evaluation frameworks within routine practice to support continuous learning and adaptation.

## Electronic supplementary material

Below is the link to the electronic supplementary material.

Supplementary Material 1

Supplementary Material 2

## Data Availability

The dataset generated and analysed during the current study are not publicly available due to privacy and ethical restrictions (i.e. potential for breach of anonymity); but are available from the corresponding author upon reasonable request.

## Abbreviations

CFIR: Consolidated Framework for Implementation Research
COREQ: Consolidated Guidelines for the Reporting of Qualitative Data
EQUITy: Enhancing the quality of psychological interventions delivered by telephone
IAPT: Improving Access to Psychological Therapies
LEAP: Lived Experience Advisory Group
MRC: Medical Research Council
NHS TTad: NHS Talking Therapies for anxiety and depression services
NIHR: National Institute of Health and Care Research
NPT: Normalisation Process Theory
RCT: Randomised Control Trial
