# Supplementary material for: Practitioner perspectives on implementing a quality improvement intervention for telephone-based therapy in NHS Talking Therapies: a qualitative process evaluation study
Source: BMC Health Serv Res. 2026 Mar 20;26:606. doi: 10.1186/s12913-026-14330-7 (PMC13126786; doi:10.1186/s12913-026-14330-7)
Supplement: Supplementary file 1 — Supplementary Material 1 [file 12913_2026_14330_MOESM1_ESM.docx]

**Practitioner Perspectives on Implementing a Quality Improvement Intervention for Telephone-Based Therapy in NHS Talking Therapies: A Qualitative Process Evaluation Study**

**APPENDIX 1**

**EQUITy intervention**

**1. Guidelines for services, team workshop and follow-up meetings**

Online day workshop for all members of the team. The morning session outlined the aims and expectations of the day, provided an overview of the EQUITy programme and telephone-delivery related policy and evidence. A Q&A session with the Programme Manager was during the session to address any queries relating to the research-related components of the trial.

The afternoon session acquainted attendees with the service guidelines developed from studies involving key stakeholders (patients, practitioners and key informant professionals) in early programme work to identify low, medium and high priority areas for their service to enhance telephone-delivered therapies. Five key areas were addressed in the guidelines: 1) Promoting Telephone Work, 2) Key elements of Telephone, Work 3) Working Environment and Resources, 4) Boosting Telephone Skills, and 5) Promoting Reflection.

The guideline booklet encouraged teams to identify one or more areas that they considered needed addressing in their service. Each area included an action plan form to assist teams to think about the steps and resources that would be needed to implement changes, identify potential barriers or challenges that may be faced and how they could be overcome, and setting a timeline.

At the end of the workshop the team collaboratively developed an agreed action plan that they would take forward and shared it with the research team. Service teams were asked to identify an action plan lead who was offered, along with any other team members, the opportunity to attend a follow-up meeting at 6-8 weeks post-workshop and subsequent bi-monthly meetings to discuss progress and any barriers to implementation of the action plan with the process evaluation lead (HB).

**2. Practitioner telephone training**

Following the workshop, two online 3-hour training sessions held on different days were provided. The sessions were aimed at practitioners who support patients over the telephone, but all team members were welcome to attend. Training sessions focused on telephone skills development, including a mixture of interactive presentations, practical small group activities, audio/visual clips and live demonstrations of good practice.

Workshops and training sessions were delivered by academic researchers with clinical backgrounds in mental health and psychology, a service-user researcher, supported by other mental health researchers with relevant experience.

**3. Resources for patients**

Patient resources (leaflet, appointment card and poster) were co-designed and developed with the EQUITy Lived Experience Advisory Group (LEAP). They addressed issues and misconceptions about telephone treatment identified in early programme studies with patients and professionals, aiming to better help patients to understand and engage with, telephone therapy.
