# Supplementary material for: Practitioner perspectives on implementing a quality improvement intervention for telephone-based therapy in NHS Talking Therapies: a qualitative process evaluation study
Source: BMC Health Serv Res. 2026 Mar 20;26:606. doi: 10.1186/s12913-026-14330-7 (PMC13126786; doi:10.1186/s12913-026-14330-7)
Supplement: Supplementary file 2 — Supplementary Material 2 [file 12913_2026_14330_MOESM2_ESM.docx]

**Enhancing the quality of psychological interventions delivered by telephone (EQUITy):**


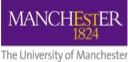

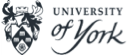

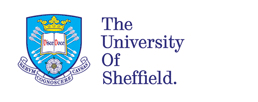

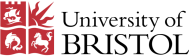

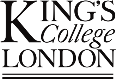

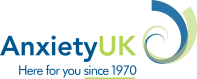

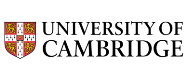


**A cluster randomised trial of a service quality improvement intervention**

**Interview-Discussion Group Schedule - Professionals**

Using the CFIR and NPT concepts to explore the EQUITy intervention: A process evaluation of the implementation for a cluster-randomised controlled trial of a complex intervention

| **Interview objectives:**   1. To explore the impact of the EQUITy intervention (i.e. Service Guidelines & Team Workshops, Telephone Training and Patient resources) on professional development, patient experience and service performance. 2. To explore what and how the EQUITy intervention components have precipitated changes in service telephone delivery. 3. To identify barriers and facilitators to the uptake and implementation of the intervention components and subsequent maintenance of changes in routine clinical practice. |
| --- |

This is an interview guide including questions/topics to be discussed with professionals (e.g. practitioners, clinical supervisors, service leads) in an individual interview or group discussion. This guide will be piloted and adapted for different service settings. Please note that the guide could be slightly modified over time following preliminary analysis during data collection.

Participants should have completed the Consent Form prior to the interview/group discussion.

**Preamble**

Reminder of the purpose of study

Explanation of ethics, consent and confidentiality of interview and analysis

Structure of the interview (may be some overlap in questions and responses)

If Group discussion ensure understanding of ***group discussion ground rules***:

- Please speak one at a time
- Be respectful of each other
- Remember: Every contribution is valuable
- Respect others’ ideas and opinions
- Keep what is said in the room confidential
- Limit the amount of identifiable information discussed (e.g. trying not to list place names and names of people)
- Breaking confidentiality if disclosures are made
- Please turn off mobiles or put on silent
- Let us know if you need to take a break

- If the group discussion is conducted remotely, specify the different ways to participate during the meeting (e.g. verbally speaking, using the chat, using the “hand” icon whenever you would like to say something)
- Anything that we’ve missed?

**Recorder turned on**

| 1. **Exploring understanding and motivation towards the implementation of the EQUITy Telephone intervention** |
| --- |
| Why did your service decided to get involve in the EQUITy trial? (question for service leads/managers) |
| What is your understanding of the EQUITy Telephone intervention?  How was the EQUITy intervention different from what you were already doing routinely? |
| (pending on response)  Remind interviewees about the different components of the EQUITy Telephone Intervention:  *-Service guidelines booklet*  *-Initial team workshop (1 full day)*  *-Follow-up team workshop (meeting/phone calls)*  *-Telephone training (2 skills sessions of 3 hours each)*  *-Patient resources (leaflet, appointment card, poster)*  Indicate with which of the components you were actively involved (e.g. attended telephone training, attended initial team workshop).  [*Remind/prompt here if not all components were discussed previously in the interview*]  Explore experiences/thoughts of the different components of the EQUITy intervention (e.g. training sessions, workshop, follow-up phone calls, patient materials)  *[Prompt: user-friendly, content, timing, enjoyed attending]*  Explore whether you feel each of the components are equally important  - |
| How would you describe your level of motivation with the implementation of the EQUITy telephone intervention?  [*Prompt: What were your thoughts/beliefs after receicing the EQUITy intervention? Where you motivated/apprehensive to implement changes in your daily practice/service? Please expand.]*  How would you describe the level of motivation of your service with the implementation of the EQUITy telephone intervention?  [Prompt: Have you identified an action lead to implement the EQUITy intervention? Have you been able to implement the expected changes following the action plan?] |
| After the initial workshop, how equipped/confident have you felt to implement the EQUITy Telephone intervention?  [*Prompts: Why/Why not?*] |
| How was the EQUITy Telephone intervention set-up in your service?  *(prompt: who would you say leads the implementation of the EQUITy intervention, coordinates the action steps for change, makes the decisions of what/how to implement the different elements of the intervention, who else is part of implementing the EQUITy intervention)* |
| How does the EQUITy Telephone intervention fit with your current work practices on a day to day basis?  Why do you think your service decided to get involve in the EQUITy trial?  What is the value (if any) of the EQUITy intervention to you as a practitioner, the patients and your service as a whole?  (*prompt: professional development –skiils, confidence, competency-, improving patient experiences, enhancing service performance on the delivery of treatment by telephone*). |
| 1. **Exploring –potential benefits of the EQUITy telephone intervention** |
| How do you evaluate the EQUITy Telephone -intervention both on your own and as a service/team?  How will you know if the EQUITy intervention has been beneficial/effective/made a difference?  (prompt: how would you measure the impact of the EQUITy intervention –e.g. rates of attendance to sessions, patient outcomes, professional confidence/competence) |
| Explore impact of the EQUITy telephone intervention (and each of the different components) in the following 3 areas:  **1- Service performance on telephone delivery** (e.g. improvement on guidelines, quality of delivery, practitioner uptake)  **2- Professional development on telephone treatment** (e.g. telephone skills, confidence, competency)  **3- Patient experiences on telephone treatment** (e.g. number of sessions attended, patient outcomes, completion of questionnaires, completion of homework, comparing patient’s feedback –i.e. before and after receciving the EQUITy intervention)  (prompt: depending on role, are you involved in any monitoring of how the EQUITy Telephone intervention is going?) |
| Following from the previous questions, in what ways do you think the EQUITy intervention (all or individual components) have **influenced** upon the **way** you/your team/service **deliver telephone treatment**  -How, why, by whom? |
| 1. **Exploring changes in service driven by the EQUITy telephone intervention** |
| In what ways has the EQUITy intervention **changed the way you/your team/service approach** telephone delivery? |
| -Did it align or **challenge/contradict existing practices** in your team/service? Where there any dilemmas between your usual practice and the EQUITy intervention?  -Did it **alter any central work processes**? Were there any workarounds?  -Do you envisage any future changes? Steps put in place to ensure |
| How have you tracked the progress of the implementation of the EQUITy intervention? (e.g. have you used the “action plan form” from the service guidelines booklet?) |
| Has the implementation of the EQUTITy intervention changed over time?  (prompt: What prompted this change? What have been the effects of this?) |
| 1. **Identyfing barriers and facilitators to the uptake and implementation of the EQUITy Telephone Intervention** |
| Reflecting on your personal experience of receiving the EQUITy intervention:  What are the things (if any) that facilitated/helped the implementation of the EQUITy Telephone intervention?  *Explore individual/team/organizational levels to know about the contextual issues* |
| What are the barriers or challenges to implement the EQUITy Telephone intervention? *How did you manage to overcome (or not) these challenges?*  *Explore this at an individual/team/organizational levels* |
| Are there any contextual aspects/procedures -influencing on the implementation of the EQUITy Telephone intervention? If yes, how? |
| 1. **Sustainability of changes driven by the EQUITy telephone intervention** |
| Covid has probed that we can deliver therapy remotely. How sustainable do you think the EQUITy Telephone intervention is in the long term? |
| What are the resources/action steps needed to maintain the changes over time?  (Explore environmental challenges and resources, e.g. what if practitioners work in GP Surgery clinics where face-to-face treatment is prioritized? What would be needed to sustain telephone treatment over time? |
| After the pandemic and things ‘return to normal’ do you think you will be able to keep the momentum going to continue to deliver telephone treatment? Why? Please expand . |
| 1. **Other** |
| Is there anything that you would like to mention/add that you don’t think we have covered? |
